# ECHOPAEDIA: Echography in Paediatric Patients in the Age of Coronavirus Disease 2019: Utility of Lung Ultrasound and Chest X-Ray in Diagnosis of Community-Acquired Pneumonia and Severe Acute Respiratory Syndrome Coronavirus 2 Pneumonia

**DOI:** 10.3389/fped.2022.813874

**Published:** 2022-02-28

**Authors:** Ivan Fiorito, Giulia Gori, Tiziano Perrone, Amelia Mascolo, Silvia Caimmi, Ilaria Palumbo, Annalisa De Silvestri, Mariangela Delliponti, Antonio Di Sabatino, Gian Luigi Marseglia

**Affiliations:** ^1^Department of Pediatrics, Foundation IRCCS Policlinico San Matteo, University of Pavia, Pavia, Italy; ^2^Department of Internal Medicine, Foundation IRCCS Policlinico San Matteo, University of Pavia, Pavia, Italy; ^3^Emergency Unit, Humanitas Gavazzeni, Bergamo, Italy; ^4^Unit of Clinical Epidemiology and Biometrics, Foundation IRCCS Policlinico San Matteo, University of Pavia, Pavia, Italy

**Keywords:** lung ultrasound, pneumonia, children, LUS score, SARS-CoV-2

## Abstract

**Background:**

In recent years, lung ultrasound (LUS) has spread to emergency departments and clinical practise gaining great support, especially in time of pandemic, but only a few studies have been done on children. The aim of the present study is to compare the diagnostic accuracy of LUS (using Soldati LUS score) and that of chest X-ray (CXR) in CAP and COVID-19 pneumonia in paediatric patients. Secondary objective of the study is to examine the association between LUS score and disease severity. Finally, we describe the local epidemiology of paediatric CAP during the study period in the era of COVID-19 by comparing it with the previous 2 years.

**Methods:**

This is an observational retrospective single-centre study carried out on patients aged 18 or younger and over the month of age admitted to the Paediatric Unit of our Foundation for suspected community-acquired pneumonia or SARS-CoV-2 pneumonia during the third pandemic wave of COVID-19. Quantitative variables were elaborated with Shapiro–Wilks test or median and interquartile range (IQR). Student's *t*-test was used for independent data. Association between quantitative data was evaluated with Pearson correlation. ROC curve analysis was used to calculate best cut-off of LUS score in paediatric patients. Area under the ROC curve (AUC), sensibility, and specificity are also reported with 95% confidence interval (CI).

**Results:**

The diagnostic accuracy of the LUS score in pneumonia, the area underlying the ROC curve (AUC) was 0.67 (95% CI: 0.27–1) thus showing a discrete discriminatory power, with a sensitivity of 89.66% and specificity 50% setting a LUS score greater than or equal to 1 as the best cut-off. Nine patients required oxygen support and a significant statistical correlation (*p* = 0.0033) emerged between LUS score and oxygen therapy. The mean LUS score in patients requiring oxygen therapy was 12. RCP was positively correlated to the patient's LUS score (*p* = 0.0024).

**Conclusions:**

Our study has shown that LUS is a valid alternative to CXR. Our results show how LUS score can be applied effectively for the diagnosis and stratification of paediatric pneumonia.

## Introduction

The management of paediatric lung infectious diseases has always been challenging for clinicians because of the variable clinical manifestations and overlapping symptoms and signs. Traditionally, chest X-ray (CXR) has played a crucial role in the diagnosis of respiratory diseases, specifically pneumonia. Nevertheless, the accuracy and sensitivity of this approach are moderate; moreover, the negative effects of radiation exposure due to CXR reduce its applications, especially in children (1, 2).

In recent years, and especially in times of pandemic, lung ultrasound (LUS) has gained great support from emergency departments and clinical practises. The advantages of this technique are its fast performance, portability, and ionising radiation-free. Pulmonary ultrasound is widely applied as an alternative diagnostic tool for community-acquired pneumonia (CAP) with bacterial and viral etiology, showing excellent results in the adult population (3–5). Similarly, infants and children might be considered ideal candidates for this type of exam due to their thinner chests and smaller lung volumes: indeed, in these patients, any lesions would more likely reach the pleura, allowing the linear probe to detect them (1, 2). Patients can be examined in various positions, such as supine, prone, and lateral decubitus. In small infants, an examination is more difficult due to the lack of collaboration. Therefore, some authors examine the small patient while feeding on the mother's lap or using distraction techniques. Usually, a high-frequency linear probe (10 MHz) is used. It can be placed either vertically, obliquely, or horizontally to the ribs in the anterior, lateral, or posterior thorax; it can be moved from one intercostal space to another, in the caudal or cranial, from the apexes to the costophrenic angles, to cover the entire lung surface (1). Some authors have focused on LUS application in children with promising data (1, 2, 6–14); in 2020, a consensus of experts have recognised an important role to point-of-care ultrasound, guided by symptoms and signs of the disease, in the management of bacterial or viral pneumonia in the paediatric population (15).

Moreover, more recently, LUS has shown its potential in the early diagnosis and management of patients with severe acute respiratory syndrome coronavirus 2 (SARS-CoV-2) infection (5, 16). High-resolution chest computed tomography (HRCT) is currently considered the gold standard for the identification of lung lesions in coronavirus disease 2019 (COVID-19) patients (17). However, HRCT is difficult to perform in paediatric clinical settings due not only to the risk of radiation exposure but also in consideration of a milder form of parenchymal damage in COVID-19 pneumonia in children rather than in adults, which often do not require such instrumentation to characterise its extent (18). Multiple groups have documented that LUS has comparable sensitivity to HRCT in identifying parenchymal damage and monitoring response to treatment in COVID-19 patients. These interstitial lesions appear similar to those of other coronavirus pneumonia, such as severe acute respiratory syndrome coronavirus (SARS-CoV-1) and Middle Eastern respiratory syndrome coronavirus (MERS-CoV) (19).

In the pathological lung, single or confluent vertical echogenic artifacts called B lines represent the phenomena related to the alteration of the density and geometry of the parenchyma (1, 5, 10). At the onset of the pandemic, an Italian group of ultrasound experts has developed a standardised thoracic ultrasound protocol, with extended posterior field scans, for the assessment and prognostic stratification of COVID-19 patients (5). The total score resulting from this protocol (LUS score) provides a quick evaluation of lung aeration, which allows defining the degree of severity of the parenchymal damage. The LUS score appears to correlate well with the pulmonary changes found in computed tomography (20); moreover, it has been shown that the LUS score correlates positively with the need for oxygen support and negatively with the oxygen saturation in the ambient air, making it extremely useful for the clinical management of patients (15).

The Soldati protocol has been widely applied in adults, yet few studies have been described in the paediatric population (21–23). Therefore, the primary aim of the present study is to compare the diagnostic accuracy of LUS (using Soldati LUS score) and that of CXR in CAP and COVID-19 pneumonia in paediatric patients. The secondary objective of this study is to examine the association between LUS score and disease severity. Finally, we describe the local epidemiology of paediatric CAP during the study period in the era of COVID-19 by comparing it with the previous 2 years.

## Materials and Methods

### Study Design

This is an observational retrospective single-centre study. We enrolled patients aged 18 years or younger at the time of admission to the Paediatric Department of the IRCCS Policlinico S. Matteo di Pavia Foundation (Lombardy, Northern Italy) for suspected community-acquired (bacterial/viral) pneumonia or SARS-CoV-2 pneumonia. Confirmation of the etiology was obtained by polymerase chain reaction on a nasopharyngeal swab for COVID-19 pneumonia, whose negativity led to the diagnosis of suspected CAP. In the period of January to May 2021 (third pandemic wave in Italy), the patients underwent a pulmonary ultrasound and chest X-ray at a maximum time difference of 24 h from each other. During the ultrasound, the LUS score, as well as the presence of pleural effusion and any irregularity of the pleura, was assessed (not such as to confer the attribution of a positive score). During the hospitalisation, patients were also subjected to blood chemistry tests, with particular attention to the inflammation indices.

The Institutional Ethics Committee has approved this study (protocol number 20210075106). Being a retrospective study, informed consent of each patient was not necessary for inclusion in the study; instead, the consent related to privacy signed at hospital admission was considered sufficient. Patient data were used in a totally anonymous form.

### Patients

Pneumonia was diagnosed following British Thoracic Society guidelines (24). All children hospitalised for signs and symptoms suspicious for pneumonia (fever, tachypnea, chest pain, intercostal re-entry, and feeding difficulties) underwent anamnesis and clinical evaluation, including vital signs. Laboratoristic evaluation included blood tests (blood count with formula) and phlogosis indices, instrumental evaluation included anteroposterior CXR and LUS, and therapy included oxygen support and eventually antibiotics. For the purpose of the study, the paediatric radiologist performing anteroposterior CXR and the operator conducting LUS were blinded to the LUS and the CXR results, respectively. The diagnosis was made during the hospitalisation based on clinical and instrumental findings. The treatment used for CAP followed the British Thoracic Society protocol; no particular protocols were performed for SARS-CoV-2 pneumonia.

### Inclusion Criteria

All children were admitted to the Paediatric Department of the IRCCS Policlinico S. Matteo di Pavia Foundation for suspected pneumonia (community-acquired or SARS-CoV-2) based on anamnestic history, clinical evaluation, blood tests, findings of the CXR, and the LUS. The final number of patients analysed in the study was 33. Of these, in four cases, the diagnosis of pneumonia was not confirmed at the time of discharge.

### Exclusion Criteria

Patients with underlying conditions such as respiratory tract abnormalities, congenital heart abnormalities, prematurity (<32 weeks gestation in children up to 2 years of age), and interstitial lung disease were excluded from the study.

### Performance of Lung Ultrasound

A GE Logiq 5 Pro ultrasound system with linear probe (5–13 MHz) and setting for the study of the thyroid was used. A maximum depth of 8 cm was set, and the single focus was positioned on the pleural line. The choice of linear vs. convex probe was made in accordance with the reduced thoracic size of the paediatric vs. the adult cohort. The mechanical index was set at 0.7 and the gain below 50% to avoid saturation phenomena. Because LUS is an artifact-based study, all cosmetic filters were avoided. Each patient underwent a systematic evaluation of the lung, according to the standardised protocol proposed by Soldati et al. (5), consisting of a sequence of 14 scans in anatomical landmarks of the chest, using intercostal scans, to cover the largest possible surface with a single scan. The reference points ranging from 1 to 6 refer to the dorsal areas. The reference points ranging from 7 to 10 refer to the side areas. Finally, the reference points between 11 and 14 refer to the anterior chest wall. In infants younger than 12 months, 10 total scans were recorded; the right and left upper middle axillary scans and right and left middle posterior axillary scans were eliminated due to the operator's lack of expertise in little children. A 5-s video clip was recorded for each scan in reference point to document the degree of pulmonary involvement. All LUS scans were performed by a single operator during hospitalisation independently of the outcome of the chest X-ray. The subsequent revision was performed by a second operator, unaware of the clinical outcome. A severity score from 0 to 3 was reported for each scan. The score of each area corresponding to a reference point was recorded, and a total LUS score was calculated by adding the individual scores. The pleural effusion and irregularities were also evaluated. The alterations did not confer a positive score but were still evident, specifically in the context of a paediatric patient's healthy lung parenchyma. The frequency of pleural effusion in CAP and SARS-CoV-2 pneumonia was calculated.

### Chest X-Ray Image Analysis

The chest radiographs were analysed by radiologists with at least 5 years of experience in paediatric thoracic radiology, unaware of the ultrasound findings found. The radiographic findings compatible with the diagnosis of pneumonia were various “consolidations, infiltrates, peribronchial or interstitial thickening.”

### Treatment

We considered the standard of care (SOC) to be the first line of treatment according to international guidelines (amoxicillin, amoxicillin/clavulanate, and ceftriaxone). In a few cases, it was necessary to resort to oxygen therapy. None of the patients required ICU admission.

### Objectives

- *Primary aim*

° To evaluate the diagnostic accuracy of LUS and CXR in identifying subjects with pneumonia.

- *Secondary aims*

° To evaluate the correlation between LUS score and the need for oxygen therapy as an index of clinical severity.
° To correlate the LUS score with bio-humoral parameters [regional cerebral perfusion (RCP), lactate dehydrogenase (LDH), and white blood count].
° To compare the LUS score obtained in pneumonia by different etiological agents (CAP *vs*. SARS-CoV-2).
° To examine the frequency of pleural effusion and pleural irregularity in CAP and SARS-CoV-2 pneumonia.
° To describe the local epidemiology of pneumonia during the observation period compared with the previous 2 years.

### Statistical Analyses

Quantitative variables were described as mean and standard deviation (SD) if normally distributed (Shapiro–Wilks test), with median and interquartile range (IQR), otherwise. They are compared between groups with Student's *t*-test for independent data. Association between quantitative data was evaluated with Pearson correlation. Receiver operating characteristic (ROC) curve analysis was used to calculate the best cutoff of LUS score in paediatric patients. The area under the ROC curve (AUC), sensibility, and specificity are also reported with a 95% confidence interval (CI).

## Results

During the third pandemic wave, a total of 33 patients admitted with suspected pneumonia were retrospectively enrolled for the study. Patients were divided into a group of confirmed cases (*N* = 29); this group included 11 SARS-CoV-2-associated cases of pneumonia and 18 cases of CAP and a group of unconfirmed cases older than 1 year, in which pneumonia was excluded (*n* = 4). The clinical features of paediatric patients are displayed in the following table (Table 1). In this study, 19 patients were male (19/33, 57%). Patient ages ranged from 2 months to 14 years 4 months, with a median age of 2 years and 4 months. The clinical manifestations included fever, cough (90% in SARS-CoV-2 population, 83% in the CAP patients, and 25% in the unconfirmed cases), chest pain (36% in SARS-CoV-2 population, 11% in the CAP patients, and 25% in the unconfirmed cases), poor feeding (18% in SARS-CoV-2 population, 33% in the CAP patients, and 25% in the unconfirmed cases), and respiratory distress (18% in SARS-CoV-2 population, 45% in the CAP group, and 25% in the unconfirmed cases group). All patients were treated with empirical antibiotic treatment. About the need for oxygen, in our cases, only low-flow oxygen was administered; the percentage of cases was 9% with an average of 4 days in SARS-CoV-2 patients, 27% with an average of 2.5 in the CAP group, and no need in the unconfirmed cases.

**Table 1:** General and clinical characteristics of population.

**Group**	**SARS-CoV-2**	**Cap**	**Unconfirmed Cases**
Age (range)	2 month−14 years	2 month−10 years	8 month−8 years
Male (% of cases)	55%	62%	50%
Days of hospitalisation (mean value)	4.5	5	4
Days of fever	2.5	3	1
Respiratory distress (% of cases)	18%	45%	25%
Cough (% of cases)	90%	83%	25%
Chest pain (% of cases)	36%	11%	25%
Poor feeding (% of cases)	18%	33%	25%
Antibiotic therapy (% of cases)	100%	100%	100%
Need for oxygen therapy (% of cases)	9%	27%	0%
Days of oxygen therapy (mean value)	4	2.5	0

**Figure 1:**
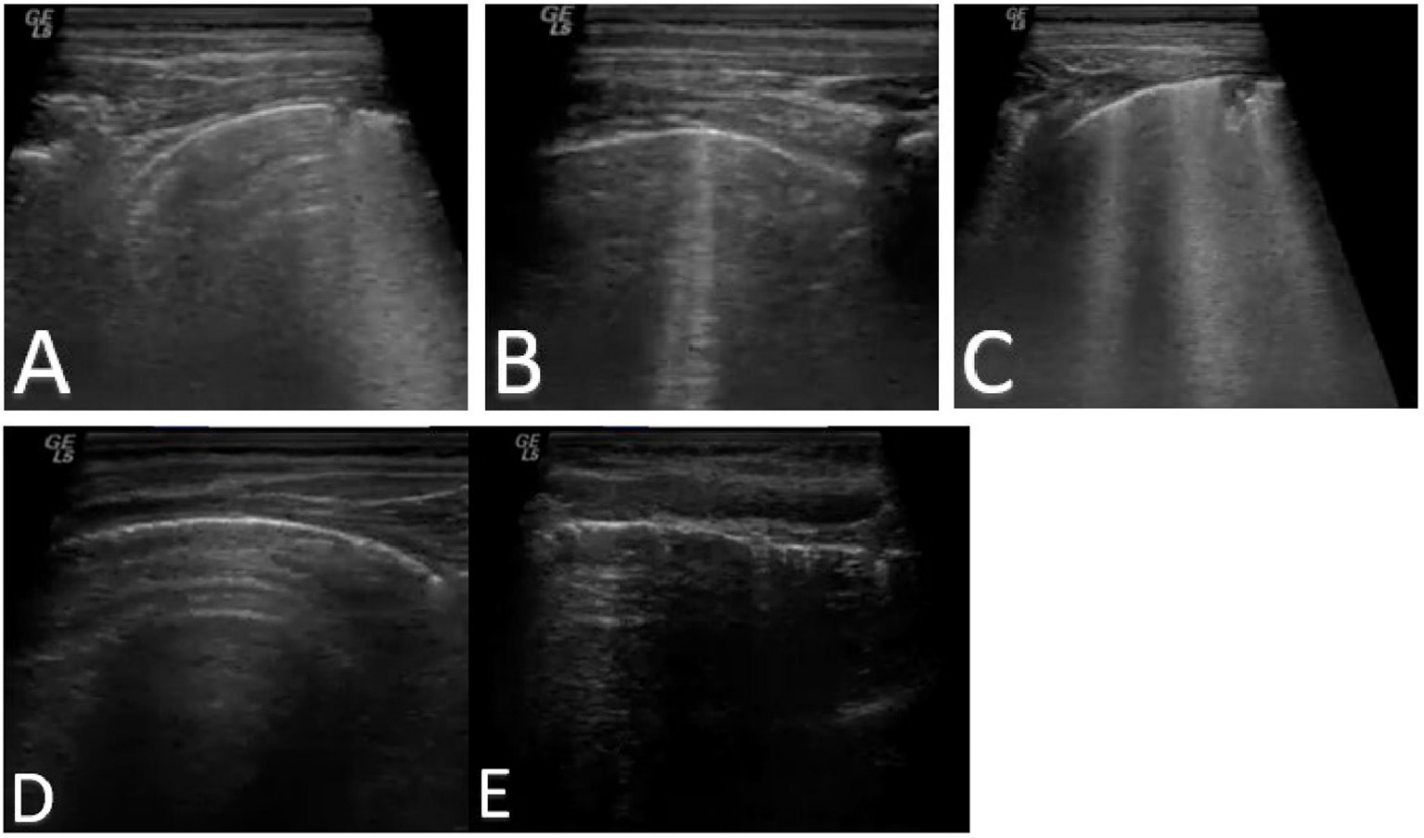
LUS score 1 **(A)**, 2 **(B)**, and 3 **(C)**. Regular pleural line **(D)** compared to pleural irregularity **(E)** in patient with COVID-19 infection.

**Figure 2:**
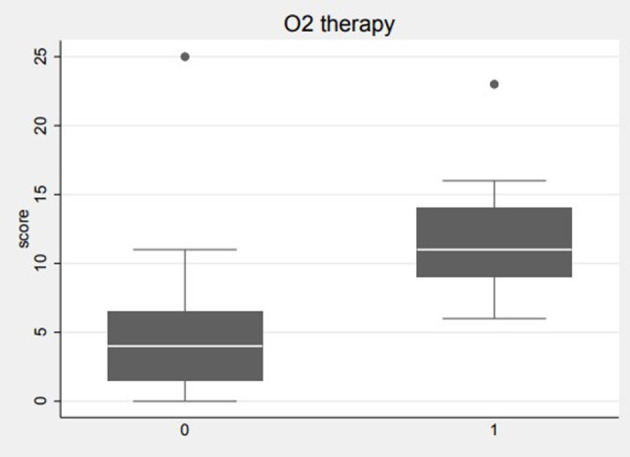
Correlation between LUS score and oxygen therapy. The average LUS score in patients requiring oxygen therapy is equal to 12.

The ultrasound analysis conducted on paediatric patients showed a median value of LUS score 6, with p25 of 2 and p75 of 9, in cases of confirmed pneumonia. Considering the diagnostic accuracy of the LUS score in pneumonia, the AUC was 0.7 (95% CI 0.27–1), thus showing a discrete discriminatory power, with a sensitivity of 89% and a specificity of 50%, setting an LUS score ≥ 1 as the best cutoff. With regard to radiography, the tool was moderately accurate, being the AUC value of 0.71 (95% CI: 0.42–1), with a sensitivity of 93.1% and a specificity of 50%. Of the 29 patients with a confirmed diagnosis of pneumonia, the radiograph was positive for consolidation compatible with pneumonia 27 times (93% of cases), whereas it was false negative in two cases. In patients with a conclusively negative diagnosis for pneumonia, two of four cases had a false-positive X-ray. Of the 29 patients with a confirmed diagnosis of pneumonia, LUS was found to be positive (LUS score > or = 1) 26 times (89% of cases), whereas it was false negative in three cases. In patients with a conclusively negative diagnosis for pneumonia, two of four cases had a false-positive LUS. Comparing the two instrumental methods, CXR and LUS were found to be concordant in identifying 24 cases of pneumonia of the total 29 affected patients. LUS identified two cases of pneumonia not detected by CXR (the total number of patients affected by pneumonia identified with LUS was 26). CXR identified three cases of pneumonia not detected by LUS (the total number of patients affected by pneumonia identified with CXR was 27). In all three of these cases, however, pleural irregularities were reported at the LUS (Figure 1). Regarding the use of oxygen therapy (low-flow oxygen therapy) in the cohort enrolled for our study, nine patients required oxygen support; moreover, a significant statistical correlation (*p* = 0.0033, *r* = 0.5) emerged between the LUS score and the need for oxygen therapy. The mean LUS score in patients requiring oxygen therapy was 12; the SD was 5 (Figure 2).

**Figure 3:**
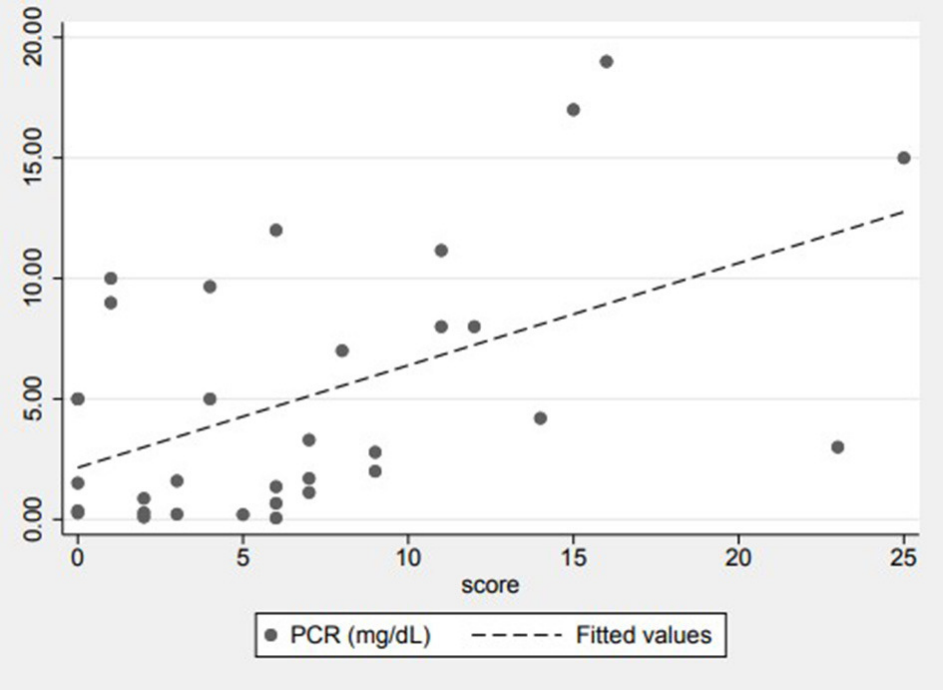
Correlation between C-reactive-protein levels and LUS score.

In evaluating the relationship between the LUS score and bio-humoral parameters indicative of inflammation (RCP, LDH, and leukocytes), no strong correlation was found except for the RCP, which is positively correlated to the patient's LUS score (*p* = 0.0024) (Figure 3). The LUS score of CAP pneumonia was higher than that found in pneumonia associated with SARS-CoV-2 infection (mean value 9 vs. 4, with an SD of 6.8 and 3.5, respectively, *p* = 0.03). Pleural effusion was found in five cases of 18 CAP; the data were confirmed only in one case by radiography due to the modest quantity of liquid present. In our paediatric cohort, no pleural effusion occurred in a patient with SARS-CoV-2 pneumonia. The general and clinical characteristics of the study population are described in Table 1. Table 2 summarises the statistical data of the two groups.

**Table 2:** Statistical data of the two groups.

**Confirmed Suspected Pneumonia**
Median LUS SCORE	6
AUC ultrasound	0.7
Sensitivity (LUS SCORE > or = 1)	83%
Specificity (LUS SCORE > or = 1)	50%
LUS score positive	26/29 (89%)
LUS SCORE in CAP (mean value, *T*-test)	9
LUS SCORE in SARS-CoV-2 (mean value, *T*-test)	4
Pleural effusion in CAP (ultrasound)	5/18 (27%)
Pleural effusion in SARS-CoV-2 pneumonia (ultrasound)	0/11 (0%)
Pleural irregularity (ultrasound)	3/29 (10%)
CXR positive	27/29 (93%)
Concordance between CXR and LUS	24/29 (82%)
**Other Diagnosis**
Median LUS SCORE	1.5
CXR positive	2/4 (50%)
LUS SCORE positive	2/4 (50%)

## Discussion

The major health challenge related to the COVID-19 pandemic and the consequent pressure on the public health system have awakened the interest in the use of the LUS (3, 5, 23). The ultrasound images collected were subsequently reexamined by two different doctors with long experience in LUS. The image acquisition protocol was developed during the COVID-19 pandemic. This is extremely valid considering the excellent correlation between ultrasound and tomodensitometric findings (the current diagnostic gold standard for pneumonia) (7, 18). Furthermore, recent works show an excellent correlation between LUS and histopathological changes in SARS-CoV-2 pneumonia (25). During the COVID-19 pandemic, several authors applied the LUS score in the paediatric setting (20, 21, 23). As suggested by Norbedo et al. (4), the LUS findings in COVID-19 pneumonia of the paediatric patient are similar to those of the adults (5). In light of the promising data from previous works, and in consideration of the pandemic period in which this work was conducted, we decided to apply the protocol and the LUS score of the study of SARS-CoV-2 pneumonia in adults in both CAP and SARS-CoV-2 pneumonia without distinction. This is the first use of this model in paediatric CAP. The main objective of this study was to evaluate the diagnostic accuracy of LUS and CXR in pneumonia of different aetiology (CAP and COVID-19) and compare the two instrumental tests. Compared with previous literature, both tools overall showed decent sensitivity in the diagnosis of pneumonia and a substantially comparable accuracy (CXR sensitivity vs. LUS, respectively, 93 and 89%, specificity 50% for both), although the small population size affected the statistical examination (2, 6, 15). This result was obtained by setting an LUS score ≥ 1 as the best cutoff. Diagnostic accuracy (particularly accuracy in diagnosing CAP) for both LUS and CXR was affected by the presence of a false-positive case. Specifically, a child was diagnosed with a paraesophageal hernia during hospitalisation, a condition causing lung compression atelectasis; this gave instrumental results compatible with lung thickening, resulting in a high LUS score (4) and paracardiac thickening CXR (26, 27). The case previously described significantly altered the diagnostic sensitivity and specificity results of both methods, given the small population examined. Table 3 shows the comparison of LUS and CXR results in the non-confirmed cases of suspected pneumonia. LUS and CXR confirmed 24 cases of pneumonia at discharge, reaching a good agreement. We examined the cases in which the two diagnostic methods were discordant: in two patients, pneumonia was confirmed by LUS, but no changes were identified on radiography, suggesting a limit for CXR in detecting small pneumonia. In both cases, pulmonary involvement was modest, with a total LUS score of 3–4 and no areas with an LUS score > 1. In three patients, pneumonia was confirmed only by chest radiography, whereas the LUS score was 0. In two of these three cases, the consolidations were detected by CXR in regions that cannot be explored with ultrasound (one retrocardiac and one subscapular); in the other case, a diffused interstitial thickening was present at CXR, but only pleural irregularity was evident at LUS. Table 4 shows the comparison of LUS and CXR results in discordant cases. These examples highlight the limitations of the ultrasound method, which presents poor accessibility to certain regions of the lung for anatomical reasons (1, 4). Interestingly, all of these three patients had mild SARS-CoV-2 pneumonia, and although not a positive LUS score was determined, LUS revealed diffuse pleural irregularities. This result shows how pleural irregularity, frequently found in healthy adult populations, can be considered an initial sign of pulmonary involvement in SARS-CoV-2-associated pneumonia, as the lung in paediatric patients has generally been subjected to less or no damage compared to adults. On the basis of these findings, more careful consideration should be given of isolated pleural irregularity as a SARS-CoV-2 ultrasound pattern of very mild lung involvement in children (22, 23). Interestingly, some authors have recently suggested a promising role of pleural irregularity in the diagnosis and follow-up of an adult patient with COVID-19 pneumonia (7). In addition, our study examined the correlation between the LUS score and the need for oxygen therapy as an index of clinical severity. There was a statistically significant correlation between LUS score and oxygen requirement with a mean LUS score in patients requiring oxygen therapy of 12; the SD was 5. In our paediatric population, only 1 case of 11 of SARS-CoV-2 pneumonia required oxygen support. The other eight cases in oxygen therapy were CAP (8/18). This result is interesting not only because it shows how the LUS score is reliable and effective in determining the degree of severity of pneumonia but also because it confirms the results of other studies; the occasional presentation of SARS-CoV-2 paediatric pneumonia with severe involvement pulmonary, although a more modest form than that which occurs in paediatric CAP, is common (18). Previous work on paediatric cohorts has not shown a correlation between ultrasound results and severity in SARS-CoV-2 pneumonia (4, 21, 22, 28). In our cohort, we report that pleural effusion was found in five cases of CAP, not associated with COVID-19-related pneumonia. Pleural effusion observation is not part of the original protocol developed by Soldati et al. (5); we used it in our study, as this was developed during the SARS-CoV-2 pandemic, where pleural effusion appears to be uncommon. Conversely, the presence of pleural effusion often appears to be associated with more severe lung involvement (29). This represents a limit to the application of the protocol in CAP, where pleural effusion is certainly more frequent and worthy of consideration. Lastly, we subsequently examined the relationship between the LUS score results and the bio-humoral parameters associated with the inflammatory condition (RCP, LDH, and leukocytes). The correlation emerged only for the C-reactive protein value, whereas it was not described for the other parameters considered, highlighting mainly the inflammatory nature of pneumonia. Regarding therapy, even patients with SARS-CoV-2 pneumonia were prudentially treated with empiric antibiotics, considering the risk of possible bacterial coinfection. Finally, our study group analysed the local epidemiology of pneumonia admitted to our Paediatric Department during the COVID-19 pandemic. In our population, during the period examined, of 18 cases of CAP, 10 were of unidentified etiology, 3 were caused by *Mycoplasma pneumoniae*, 2 were associated with rhinovirus, and 3 with adenovirus. In 2021, admissions to our foundation for pneumonia decreased by approximately two-thirds compared with the previous 2 years (2019–2020). Specifically, considering the same time of the year (January to May), in 2019, 72 patients were hospitalised with a diagnosis of pneumonia, of which 18 associated with confirmed viral infections (adenovirus, rhinovirus, respiratory syncytial virus, and influenza virus A), 9 from bacteria (*M. pneumoniae*), and the remaining cases from an unidentified cause. In 2020, 70 patients were hospitalised for pneumonia, of which 22 cases were associated with bacterial infection (*M. pneumoniae*), 3 cases were associated with viruses (influenza A virus), and 5 were associated with SARS-CoV-2; in the remaining cases, the causative agent was not identified. In 2021, the reduction in hospitalisations for pneumonia was certainly partly attributable to the measures implemented to prevent and contain the spread of SARS-CoV-2 disease (30). During the COVID-19 pandemic, and in consideration of the high percentage of pneumonia associated with it (and with other viruses), the application of a single image acquisition protocol will be of great interest considering that at the moment, there is no peculiar pattern permitting to distinguish between different viruses (9), moreover, if the technique is able to accurately analyse the extent of pulmonary involvement and to provide for proper clinical-therapeutic management by identifying the need for oxygen. Our study is only a small experience, and the data will need to be confirmed by larger studies.

**Table 3:** Comparison of LUS and CXR results in the cases with other diagnosis.

**Other Diagnosis**			
**Patients**	**Total LUS Score**	**CXR**	**Diagnosis**
Patient n.1	15	Positive	Paraesophageal hernia
Patient n.2	1	Negative	SARS-CoV-2 infection
Patient n.3	0	Negative	SARS-CoV-2 infection
Patient n.4	0	Positive	Bronchitis

**Table 4:** Comparison of LUS and CXR results in the discordant cases.

**LUS-CXR: Discordant Cases**
**Patients**	**Total LUS Score**	**CXR**	**Pleural Irregularity**	**Diagnosis**
Patient n.1	0	Paracardiac consolidation	Present	SARS-CoV-2 pneumonia
Patient n.2	0	Interstial thickening	Present	SARS-CoV-2 pneumonia
Patient n.3	0	Subclavear consolidation	Present	SARS-CoV-2 pneumonia
Patient n.4	3	0	Absent	SARS-CoV-2 pneumonia
Patient n.5	4	0	Absent	CAP

## Conclusion

This is a retrospective and monocentric study carried out on a cohort of 33 children admitted to the paediatric ward for suspected community-based or SARS-CoV-2-related pneumonia during the third wave of the COVID-19 pandemic in Italy having the intrinsic limits of a small sample size. Our intent is to encourage greater interest in the application of LUS in the paediatric field, where this is not yet widely taught and applied. Our study, in line with the data in the literature, has shown that LUS is an excellent alternative to chest radiography, with the benefit of being free from the harmful effects of radiation. Our results show how the LUS score can be effectively applied for the diagnosis and stratification of paediatric pneumonia. A score of 12 appears to be associated with severe pneumonia requiring oxygen support. The protocol used in this study, regardless of the etiology of pneumonia, could allow for a reliable assessment of pulmonary involvement, as well as a prediction of severity, and therefore clinical-therapeutic management based on the total score obtained. In this study, COVID-19 pneumonia appeared more modest than CAP with a lower LUS score, requiring oxygen support in only one case and in no case presenting with pleural effusion. These data are in agreement with the literature, as COVID-19 pneumonia is usually milder than CAP in children and rarely presents with pleural effusion, a complication that has been seen to be associated with a worse patient outcome (13, 18, 27). Finally, we describe how pleural irregularity is a recurrent pattern in COVID-19 lung infections of children in which the lung is less prone to non-specific changes found in an adult. We, therefore, suggest this to be taken into account in the image acquisition, as it could represent an early and mild form of SARS-Cov2-related parenchymal lung disease.

## Data Availability Statement

The raw data supporting the conclusions of this article will be made available by the authors, without undue reservation.

## Ethics Statement

The studies involving human participants were reviewed and approved by Ethics Committee IRCCS Policlinico San Matteo Foundation. Written informed consent to participate in this study was provided by the participants' legal guardian/next of kin.

## Author Contributions

All authors listed have made a substantial, direct, and intellectual contribution to the work and approved it for publication.

## Conflict of Interest

The authors declare that the research was conducted in the absence of any commercial or financial relationships that could be construed as a potential conflict of interest.

## Publisher's Note

All claims expressed in this article are solely those of the authors and do not necessarily represent those of their affiliated organizations, or those of the publisher, the editors and the reviewers. Any product that may be evaluated in this article, or claim that may be made by its manufacturer, is not guaranteed or endorsed by the publisher.

## Abbreviations

LUS: lung ultrasound
CXR: chest X-ray
COVID-19: coronavirus disease 2019
SARS-CoV-2: severe acute respiratory syndrome coronavirus 2
CAP: community-acquired pneumonia
HRCT: high-resolution computed tomography
CRP: C-reactive protein
LDH: lactate dehydrogenase
IQR: interquartile range
AUC: area under the curve
CI: confidence interval.
